# Toward a new cognitive neuroscience: modeling natural brain dynamics

**DOI:** 10.3389/fnhum.2014.00444

**Published:** 2014-06-19

**Authors:** Klaus Gramann, Tzyy-Ping Jung, Daniel P. Ferris, Chin-Teng Lin, Scott Makeig

**Affiliations:** ^1^Psychology and Ergonomics, Biological Psychology and Neuroergonomics, Berlin Institute of TechnologyBerlin, Germany; ^2^Center for Advanced Neurological Engineering, University of California San DiegoSan Diego, CA, USA; ^3^Institute for Neural Computation, University of California San DiegoSan Diego, CA, USA; ^4^Institute of Engineering in Medicine, University of California San DiegoSan Diego, CA, USA; ^5^Department of Computer Science, National Chiao-Tung UniversityHsinchu, Taiwan; ^6^Department of Biomedical Engineering, University of MichiganAnn Arbor, MI, USA; ^7^School of Kinesiology, University of MichiganAnn Arbor, MI, USA; ^8^Electrical and Computer Engineering, National Chiao-Tung UniversityHsinchu, Taiwan; ^9^Brain Research Center, National Chiao-Tung UniversityHsinchu, Taiwan; ^10^Swartz Center for Computational Neuroscience, University of California San DiegoSan Diego, CA, USA

**Keywords:** mobile brain/body imaging, EEG, fNIRS, brain mapping, embodied cognition, natural cognition, wireless EEG sensors, computational neurosciences

Decades of brain imaging experiments have revealed important insights into the architecture of the human brain and the detailed anatomic basis for the neural dynamics supporting human cognition. However, technical restrictions of traditional brain imaging approaches including functional magnetic resonance tomography (fMRI), positron emission tomography (PET), and magnetoencephalography (MEG) severely limit participants' movements during experiments (Makeig et al., 2009). As a consequence, our knowledge of the neural basis of human cognition is rooted in a dissociation of human cognition from what is arguably its foremost, and certainly its most evolutionarily determinant function—organizing our behavior so as to optimize its consequences in our complex, multi-scale, and ever-changing environment. The concept of *natural cognition*, therefore, should not be separated from our fundamental experience and role as an embodied agent acting in a complex, partly unpredictable world.

To gain new insights into the brain dynamics supporting natural cognition requires overcoming restrictions of traditional brain imaging technologies (Gramann et al., 2011). First, the sensors must be lightweight and untethered to allow monitoring of brain activity during free movements. Fortunately, new electroencephalography (EEG) and near infrared spectroscopy (NIRS) sensors and sensing devices allow recording both electrical and hemodynamic brain and body activity while participants are freely moving (Lin et al., 2011; Liao et al., 2012; Ayaz et al., 2013). New data-driven analysis approaches must allow separation of signals arriving at the sensors from the brain as well as non-brain sources like neck muscles, eyes, heart, and the electrical environment (Makeig et al., 2004). Independent component analysis (ICA) and related blind source separation methods have proven effective for separating brain from non-brain activities from electrophysiological data recorded during experimental paradigms that stimulate natural cognition (Gramann et al., 2014). ICA has also proven valuable for separating other multi-channel signals including electromyographic (EMG) and electrocardiographic (ECG) activities (Gramann et al., 2010; Gwin et al., 2010; Kline et al., 2014).

Adequate study of natural cognition also requires synchronous recording of participants' motor actions as well as the physical environment and external events influencing cognition. Recording what the brain does (via EEG and fNIRS brain imaging), what it senses (via scene and event recording), and what it organizes (via motor, ocular, and autonomic activity recording) may be termed *mobile brain/body imaging* (“MoBI”). Technically, recording MoBI data is now possible at reasonable cost and convenience. However, joint multi-stream analysis of the data recorded in MoBI paradigms presents major conceptual, mathematical, and data processing challenges (Ojeda et al., 2014).

To overcome restrictions of established brain imaging methods and to facilitate further development of mobile brain/body imaging, a group of researchers from all over the world gathered in the beautiful scientific retreat of the Hanse-Wissenschaftskolleg in Delmenhorst, Germany in September 2013 for the first international meeting on Mobile Brain/Body Imaging. During a stimulating and intense workshop, attendees presented and discussed newest developments in mobile brain imaging technologies, novel software architectures for recording and analyzing multidimensional data streams, and other topics relevant to MoBI. Most attendees at the Delmenhorst meeting contributed to this Research Topic; other research groups have added contributions sharing related ideas. The present Research Topic thus provides an excellent overview of the current state of the art in mobile brain/body imaging. The topics cover the three main pillars of MoBI research, i.e., hardware for imaging mobile brain and body dynamics, software to record and analyze complex multi-dimensional data streams, and applications of MoBI to such diverse fields as neuroergonomics, gait rehabilitation, spatial cognition, and dance.

Starting with the technical aspects of MoBI, Reis et al. (2014), provide an overview on existing hardware and software solutions for MoBI recordings. Focusing on new sensor technology and analysis approaches, Lin et al. report a test of a new mobile EEG headgear using steady-state visual-evoked potentials in participants during treadmill walking (Lin et al., 2014). With the aim to include gaze tracking as an important information channel for investigations of natural cognition and associated brain dynamics Browatzki and colleagues describe and compare two different approaches to measuring eye movements in mobile participants (Browatzki et al., 2014).

The second pillar of MoBI, software frameworks for recording and analyses of multi-modal imaging data is addressed by Ojeda and colleagues providing a description of a new open source toolbox (Ojeda et al., 2014). MoBILAB interoperates with EEGLAB (Delorme and Makeig, 2004) and allows for analysis and visualization of multidimensional mobile brain/body imaging data. Zao et al. (2014) introduce an exciting new perspective on distributed computing describing a novel network system approach to remote monitoring of brain/body activity of one or many mobile participants.

The majority of contributions to this Research Topic can be summarized under the pillar of MoBI applications. The review by Mehta and Parasuraman (2013) provides an overview of the advantages and disadvantages of existing imaging modalities in the area of neuroergonomics, describing differing temporal and spatial resolutions and the degree of immobility that brain imaging method imposes on participants. Ayaz et al. (2013) describe the development and application of a mobile fNIRS device for investigating changes in workload in real operating environments providing an example of mobile recordings of hemodynamics. The first investigation of kinesthetic and vestibular information processing in actively navigating participants is given by Ehinger et al. (2014). The authors dissociate the brain dynamics underlying different proprioceptive senses during movements. Wagner et al. (2014) use MoBI to describe the cortical networks activated during robot-assisted walking and investigate the potential impact of movement-related feedback for gait rehabilitation. Cruz-Garza and colleagues investigate professional dancers during different whole body movements and derive distinct expressive qualities of movement from surface EEG (Cruz-Garza et al., 2014). While the previous studies used whole body movement, Amengual and colleagues describe the brain dynamics associated with the preparation and execution of multi-joint self-paced arm movements (Amengual et al., 2014). Finally, in their paper Derix et al. elucidate the neuronal basis of mental processes during natural communication based on electrocorticography in pre-neurosurgical patients (Derix et al., 2014).

All contributions in this Research Topic go beyond the state of the art in brain imaging and provide new approaches to recording and analyzing multi-modal data. The authors describe new insights into the neural basis of cognitive processes beyond traditional laboratory research. We hope this Research Topic may inspire new research that uses the MoBI paradigm to investigate natural cognition by recording and analyzing brain dynamics and behavior of participants performing a wide range of naturally motivated actions and interactions.

## Conflict of interest statement

The authors declare that the research was conducted in the absence of any commercial or financial relationships that could be construed as a potential conflict of interest.
